# Examining the influence of health sector coordination on the efficiency of county health systems in Kenya

**DOI:** 10.1186/s12913-023-09344-4

**Published:** 2023-04-11

**Authors:** Lizah Nyawira, Rebecca G Njuguna, Benjamin Tsofa, Anita Musiega, Joshua Munywoki, Kara Hanson, Andrew Mulwa, Sassy Molyneux, Isabel Maina, Charles Normand, Julie Jemutai, Edwine Barasa

**Affiliations:** 1 Health Economics Research Unit, KEMRI-Wellcome Trust Research Programme, Nairobi, Kenya; 2 Health Systems and Research Ethics Department, KEMRI-Wellcome Trust Research Programme, Kilifi, Kenya; 3 Faculty of Public Health and Policy, London School of Hygiene and Tropical Medicine, London, UK; 4 Directorate of Medical Services, preventive and promotive health, Ministry of Health, Nairobi, Kenya; 5 Centre for Tropical Medicine and Global Health, Nuffield Department of Medicine, University of Oxford, Oxford, UK; 6 Health Financing Department, Ministry of Health, Nairobi, Kenya; 7 Centre for Health Policy and Management, Trinity College, the University of Dublin, Dublin, Ireland; 8 Institute of Healthcare Management, Strathmore Business School, Strathmore University, Nairobi, Kenya

**Keywords:** Co-ordination, Efficiency, Performance, Kenya

## Abstract

**Background:**

Health systems are complex, consisting of multiple interacting structures and actors whose effective coordination is paramount to enhancing health system goals. Health sector coordination is a potential source of inefficiency in the health sector. We examined how the coordination of the health sector affects health system efficiency in Kenya.

**Methods:**

We conducted a qualitative cross-sectional study, collecting data at the national level and in two purposely selected counties in Kenya. We collected data using in-depth interviews (n = 37) with national and county-level respondents, and document reviews. We analyzed the data using a thematic approach.

**Results:**

The study found that while formal coordination structures exist in the Kenyan health system, duplication, fragmentation, and misalignment of health system functions and actor actions compromise the coordination of the health sector. These challenges were observed in both vertical (coordination within the ministry of health, within the county departments of health, and between the national ministry of health and the county department of health) and horizontal coordination mechanisms (coordination between the ministry of health or the county department of health and non-state partners, and coordination among county governments). These coordination challenges are likely to impact the efficiency of the Kenyan health system by increasing the transaction costs of health system functions. Inadequate coordination also impairs the implementation of health programmes and hence compromises health system performance.

**Conclusion:**

The efficiency of the Kenyan health system could be enhanced by strengthening the coordination of the Kenyan health sector. This can be achieved by aligning and harmonizing the intergovernmental and health sector-specific coordination mechanisms, strengthening the implementation of the Kenya health sector coordination framework at the county level, and enhancing donor coordination through common funding arrangements and integrating vertical disease programs with the rest of the health system. The ministry of health and county departments of health should also review internal organizational structures to enhance functional and role clarity of organizational units and staff, respectively. Finally, counties should consider initiating health sector coordination mechanisms between counties to reduce the fragmentation of health system functions across neighboring counties.

## Introduction

Kenya has made a commitment to achieve Universal Health Coverage (UHC) by the year 2030 [1]. To progress toward UHC, Kenya will need to mobilize additional resources for the health sector [2]. While it has been observed that public health expenditure by low- and middle-income countries should be a minimum of 5% of their gross domestic products (GDP) to make progress towards UHC, Kenya’s level of public expenditure on health is estimated to be only 2% of GDP [3].

Improving health system efficiency is considered a potential strategy for expanding the fiscal space for health [4]. Fiscal space refers to a government’s budgetary room to apply resources without negatively impacting its financial sustainability [5]. Efficiency within the health system refers to maximizing health outputs and outcomes using available resources [6]. Two types of efficiency exist and form the overall efficiency of a health system; technical and allocative efficiency. Technical efficiency refers to achieving maximum output with the least cost while allocative efficiency refers to how different resources are combined to produce a mix of different outputs [6]. Globally, it is estimated that 20–40% of health system resources are wasted by inefficiencies [7].

Health systems consist of multiple actors whose effective coordination is paramount to enhancing health system goals [8, 9]. Poor health sector coordination has been identified as a potential source of inefficiency in the health sector [10–12]. Coordination has been defined as the extent to which organizational units or levels ensure that their activities take into account those of other units or levels [13]. Coordination thus implies the alignment of institutional arrangements, functional assignment, and implementation efforts in the health sector to avoid duplication and ensure effective use of resources. Coordination is relevant for all health system elements, such as governance, financing, human resources, human resource management, commodity supply chains, information systems, and service delivery.

Through the Kenya Efficiency Study (KES), we sought to examine the factors which influence health system efficiency in Kenya and the mechanisms through which this occurs. The study was conducted in three phases with the first phase conceptualizing efficiency and potential determinants of health system efficiency in Kenya. This was done through an extensive literature review [14] and stakeholder consultation [15]. Stakeholders identified health sector coordination as a potential factor influencing county health system efficiency in Kenya [15]. The second phase of the study used data envelopment analysis (DEA) to measure the technical efficiency of the 47 county health systems [16] and informed the selection of the counties for case studies in the third phase of the study. This paper reports findings from the third phase of the KES study, focusing on health sector coordination. While there is literature on health sector coordination, most of it has either focused on a narrow dimension -such as coordination for a specific activity (for, example human resources) for specific actor (for instance, donor coordination) or a singular dimension (such as multi-sectoral coordination). Further, there is limited literature on the interaction between health sector coordination and the efficiency of health systems. Specifically for Kenya, no prior study has examined health sector coordination within the decentralized context of the health system. This paper, therefore, aims to contribute to filling these knowledge gaps. The paper aims to investigate coordination in its multiple dimensions within the Kenyan health system rather than a singular dimension. The paper also aims to investigate how these coordination practices influence the efficiency of county health systems in Kenya.

## Methods

### Conceptual framework

**Fig. 1:**
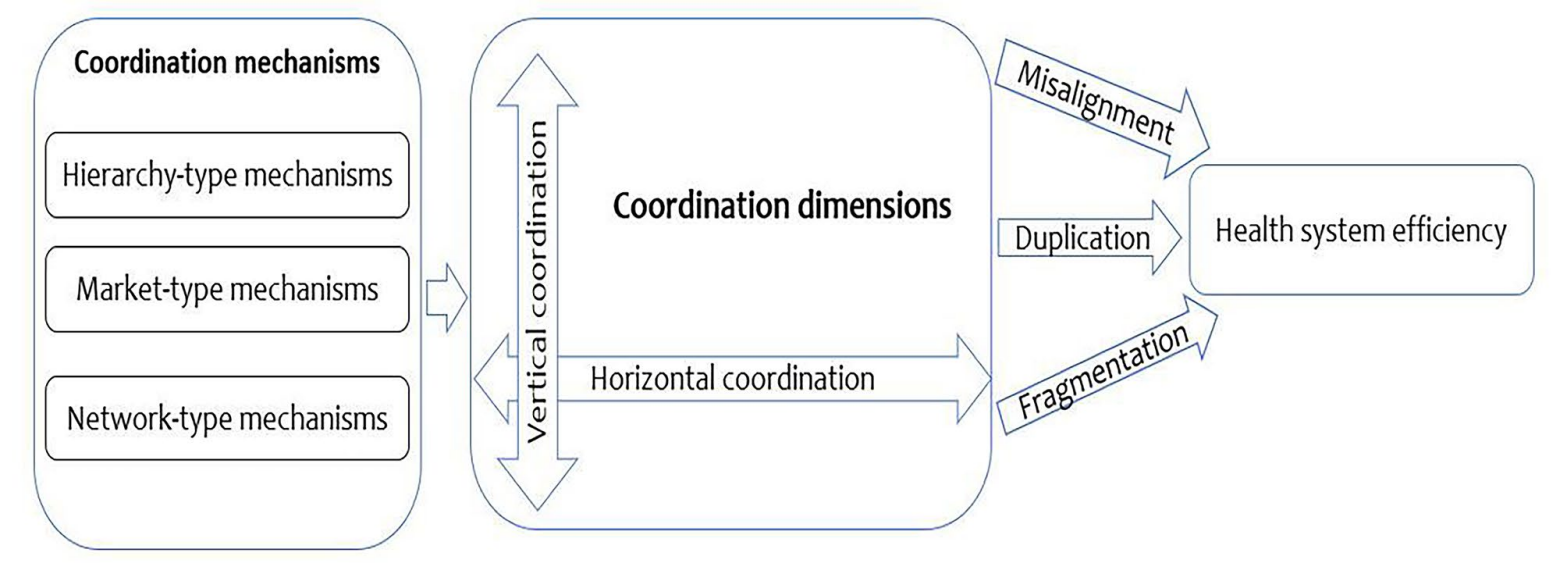
Health Sector Coordination and health system efficiency conceptual framework

We developed a descriptive conceptual framework drawing on literature about government coordination more generally and coordination in the health sector (Fig. 1). The framework focuses on coordination within government and between government and non-state actors such as donors. The framework assumes that poor coordination occurs when there is (1) misalignment, (2) duplication, or (3) fragmentation of institutional structure and implementation processes in the health sector [13, 17–19]. Misalignment occurs when multiple health system structures or processes do not yield coherent incentives and outcomes. Duplication occurs when health system actors, structures, and processes perform the same task. Fragmentation occurs when structures and processes that ought to share goals are distributed across multiple actors in ways that lead to gaps or misalignments.

Coordination occurs along two dimensions: horizontal and vertical [13]. Horizontal coordination refers to forms of coordination between organizations or units on the same hierarchical tier within government. This study includes coordination across directorates within the Ministry of Health (MoH) and across semi-autonomous government agencies (SAGA’s) at the national level. It also includes coordination between counties and across departments within the county governments. The coordination between national or county governments and non-state actors is also horizontal. Vertical coordination occurs between organizational units within an entity but at varying levels of the hierarchy or power structure. In this study, vertical coordination includes coordination between the national government and county government, between the MOH and its departments, programmes, and SAGAs, and between county departments of health and sub-county health teams.

Coordination mechanisms are classified into either or a combination of (1) hierarchy-type mechanisms, (2) market-type mechanisms, and (3) network-type mechanisms [20, 21]. Hierarchy type mechanisms rely on authority as the basic control lever and are implemented as rules and orders. This type of mechanism is hence more suited to vertical coordination. Market type mechanisms rely on the price mechanism, self-interest and incentives and are based on competition and bargaining and are typically employed to coordinate the private sector and semi-autonomous public agencies by the creation of internal markets. Network type mechanisms rely on cooperation between actors whose relationships are governed by interdependencies, trust and the responsibilities of each actor. Network type mechanisms are suited for horizonal coordination where the actors do not have authority over each other.

Coordination may affect health system efficiency through three mechanisms [22–24]. First, coordination influences transaction costs of health system implementation. Inadequate coordination in the form of duplication and fragmentation increases transaction costs leading to inefficiency. Second, inadequate coordination in the form of duplication and/or fragmentation may lead to voids of responsibility in executing some key functions [25]. This results in inadequate implementation of certain activities that are critical for health system performance, which is likely to compromise health system outcomes which in turn compromises efficiency. Third, misalignment of actions is likely to reduce the effectiveness of implementation and hence compromise health system outcomes which also compromises health system efficiency.

We applied this conceptual framework in the development of our data collection tools, and in data analysis.

### Study design

We used a qualitative cross-sectional design and collected data at the national and county level. Two counties were purposively selected based on their efficiency scores from the larger KES within which this study is nested to include one high scoring county (County A) and one low scoring county (County B). This strategy to selection of counties was informed by the hypothesis that differences in efficiency would be associated with the level of coordination in the health sector.

### Study setting

Kenya has a devolved system of governance with a national government and 47 semi-autonomous county governments [26]. The national government has policy and regulatory roles while the county governments are responsible for health service delivery, priority setting and overall management of health sector service delivery resources. There are 47 county governments in Kenya, which are administratively divided into sub-counties, and the sub-counties in turn divided into wards. Table 1 shows the characteristics of our two case studies selected based on their efficiency scores [16].

**Table 1:** Characteristics of study counties in 2019

	County A	County B
Population [27]	1,670,570	268,002
Population Density (persons/sq km) [27]	552.5	10.57
Urban population [27]	190,112	142,333
Rural population [27]	1,480,458	125,669
Efficiency score [16]	0.88	0.57

### Study population and data collection

At the national level, we collected data from the MoH, program staff, SAGAs and donors. In each study county, we collected data at the county and sub-county administration levels, and at the healthcare facility level. We selected two public healthcare facilities, a hospital (Level 4 or 5) and a primary health facility (Level 2 or 3), in each of the study counties. We selected individual facilities within the counties through random sampling from the Kenya Master Health Facility List (KHFML). We collected data using in-depth interviews (KIIs), document and secondary data reviews.

#### In-depth interviews

We carried out purposive sampling of respondents for the KIIs based on their roles and experiences at national and county levels. The inclusion criterion for the respondents was a working duration of not less than six months to ensure a relatively good understanding of the co-ordination issues within the health system. We stopped data collection at data saturation/the point where no new information was obtained after conducting additional interviews. We conducted a total of 37 interviews. All study participants were drawn from the health sector. Table 2 outlines the distribution of study respondents across the levels of the health system and study counties.

**Table 2:** Distribution of study respondents across the levels of the health system and study counties

	County A	County B
Health Facility Managers	8	4
Health Care Providers	1	1
Sub-County Managers	0	3
County Officials	4	3
Program Coordinators	3	1
Total per County	16	12
National Level	9	
Total Interviews	37	

We conducted interviews using a topic guide whose development was informed by the study’s conceptual framework. We conducted interviews at private locations in the working stations of the study respondents or an alternative location that the respondents deemed suitable and confidential. We conducted all county level KIIs through physical meetings and some national level interviews virtually due to participant preference. Each in-depth interview lasted approximately 45 to 60 min. We audio recorded all interviews using a digital recorder. We carried out peer debriefing among the researchers after each KII to enhance credibility of the data collected [28]. The researchers constantly reflected about the knowledge assumptions made in the entire process of the research and how this may affect the findings.

#### Review of documents and secondary data

We collated and analysed documents and reports containing information on or related to co-ordination within the health system (Table 3). These documents were identified using a combination of researcher knowledge, recommendations from study participants. A document was selected if it contained information about the coordination of health sector functions/actors in Kenya. We extracted relevant information from the selected documents using document review data abstraction guides and transferred these to a document review summary form. The collection of data from multiple sources (interviews, documents, information systems) facilitated data triangulation.

**Table 3:** Sources of secondary data

Data Sources	
Intergovernmental relations act [29]	
Kenya Health Sector Strategic Plan 2018–2023[30]	
Kenya Health Partnership and Co-ordination Framework [31]	
Mid-term Review of the Kenya Health Sector Strategic and Investment Plan (2014–2018) [32]	
Ministerial Strategic & Investment Plan July 2014– June 2018 [33]	
County documents such as the county integrated development plan (CIDP)	

We pre-tested data collection tools through a pilot exercise to minimize bias and enhance validity of the data collection tools.

### Data management and analysis

We transcribed interview audio files into MS Word. We cross-checked transcripts against the audio recordings as a quality assurance measure. We then imported transcribed data and then imported them into NVIVO 10 software (QSR International, Australia) for coding and to aid with the analysis. Each transcript had a unique identifier consisting of a code, date, and respondent identifier to enhance anonymity and facilitate informed analysis. We employed a thematic approach to provide interpretations and practical recommendations that will be relevant to policymakers. First, we developed a coding framework based on the conceptual framework, and preliminary emerging themes. A discussion was then held between the LN and EB to obtain consensus on the final coding framework. We then coded all transcripts and documents using the final coding framework while allowing for the emergence of new themes. We subsequently charted coded data, which entailed summarizing the findings from each transcript based on the various themes and providing illustrative quotes. Lastly, we interpreted data by identifying connections between the various themes.

### Enhancing rigor and trustworthiness

We endeavoured to enhance the trustworthiness of the study in various ways. First, the study was guided by a conceptual framework that was developed from the literature. Second, we employed method triangulation by using multiple data collection methods (interviews and document reviews) and data triangulation by collecting data from multiple participants that hold varied roles and occupy varied spaces in the health system. Third, we held peer debriefing sessions to reflect on the study findings and their interpretations. This was especially important to tackle potential biases inherent in the positionality of some of the study team members. The study team members include staff from the Kenyan ministry of health and county governments, and staff from a state corporation under the ministry of health with the mandate to carry out health research. This composition was intentional with the aim of co-producing the research between practitioners/policymakers and researchers to enhance the purposefulness of the study, ownership of the study findings, and evidence-informed policymaking. To manage the potential influence of this positionality, the study team also included members outside of these institutions, and outside of the country setting who contributed and were part of peer debriefing sessions.

### Ethical considerations

We obtained ethics review and approval (KEMRI/SERU/CGMR-C/154/3814) for this study from the KEMRI Scientific and Ethics Review Unit (SERU). We obtained approvals from other relevant authorities prior to commencement of the study. We provided all study participants with written informed consent to participating in the study and being audio recorded. Confidentiality was assured by anonymizing the study counties, de—identification of respondent data, securing the collected data in password protected computers, and restricting access to the data to research participants only.

## Results

In this section we begin by describing the formal health sector coordination arrangements, followed by a presentation of findings on health sector coordination at the national MOH level, the county department of health level, and between donors and the government (national and county level).

### Formal health sector coordination mechanisms in the kenyan the health sector in theory

At the national level, the MOH is administratively organized into 6 directorates [34], and also provides oversight for 7 semi-autonomous government agencies (SAGAs) and 8 regulatory bodies (Table 4) [35]. Additionally, the National Spinal Injury Hospital, Mathari National Teaching and Referral Hospital, National Reference Laboratories and Government Chemist are also under the mandate of the MoH [35]. The directorates are comprised of departments, which are comprised of divisions, which are in turn comprised of units, each responsible for a specific function.

At the time of collecting the data, the MOH was headed by the Cabinet Secretary (CS) who oversees the overall operations of the ministry, and who is assisted by two Chief Administrative Secretaries (CAS). The accounting officer for the MOH is the Principal Secretary (PS). The technical leadership of the MOH is provided by the Director General (DG) of health, who provides oversight and supervision of the heads of the technical directorates.

**Table 4:** List of SAGAs and regulatory bodies and their key mandate

**SAGA**
Kenyatta National Hospital (KNH)
Moi Teaching and Referral Hospital (MTRH)
Kenya Medical Training College (KMTC)
Kenya Medical Research Institute (KEMRI)
Kenya Medical and Supply Agency (KEMSA)
National Health Insurance Fund (NHIF)
National Aids Control Council (NACC)
**REGULATORY BODIES**
Kenya Medical Practitioners and Dentists Council (KMPDC)
Clinical Officers Council (COC)
Kenya Medical Laboratory Technicians and Technologists Board (KMLTTB)
Nursing Council of Kenya (NCK)
Kenya Nutritionist and Dietetics Institute (KNDI)
Public Health Officers and Public Health Technicians Council
Pharmacy and Poisons Board (PPB)
Radiation Protection Board

County governments are organized into departments, that are each headed by a member of the county executive committee (CEC). The county department of health (CDOH) is thus headed by the CEC member for health (CEC Health). The accounting officer for the CDOH is the chief officer, while the director of health provides technical leadership of the department. At the county level, the heads of different divisions within the CDOH constitute the county health management team (CHMT), while their equivalent at the sub-county level are the sub-county health management teams (SCHMT). The CHMT, and SCHMT are responsible for planning, budgeting and coordinating the implementation of health sector activities at the county and sub-county level respectively.

Coordination mechanisms within the Kenyan health sector are mainly hierarchical and network-type in nature. Within the context of devolved governance, Kenya has an inter-governmental relations law that establishes a vertical coordination framework between the national and county governments, and horizontal coordination among county governments [29]. This law establishes a national and county government coordinating summit, a hierarchical mechanism, which is the highest body for coordinating intergovernmental relations. The summit comprises of the President of the republic of Kenya, and the governors of the 47 counties. The law also establishes a Council of Governors (COG), a network-type mechanism, that comprises of the 47 county governors and coordinates county government activities. The COG has sector specific committees, including health, and committee chairs drawn from among the 47 governors.

To execute the technical mandates of the Summit and the COG, the law establishes an intergovernmental relations technical committee that comprises of members and a secretariat that are competitively recruited by the summit. The technical committee has sector specific committees including a health committee. Among others the health committee coordinates health sector specific intergovernmental relations (national and county, and among counties).

Within the Kenyan health sector, coordination is formally guided by the Kenya health sector partnership and coordination framework which employs hierarchical mechanisms to coordinate actors within government and network-type mechanisms to coordinate state and non-state actors [31]. This framework adopts a sector-wide approach (SWAp) and aims to (a) reduce transaction costs to the government and other actors, (b) develop collaborative relationships between actors that will enhance efficiency and effectiveness, (c) facilitate coordination of funding and activities between actors to eliminate duplication of efforts, and (d) to facilitate shared accountability for results [36]. The SWAp approach was adopted in the Kenyan health sector in 2005 with the development of the national health sector strategic plan II 2005–2012. The SWAp aimed to achieve health sector coordination among partners through joint planning, joint monitoring, and joint coordination.

**Fig. 2:**
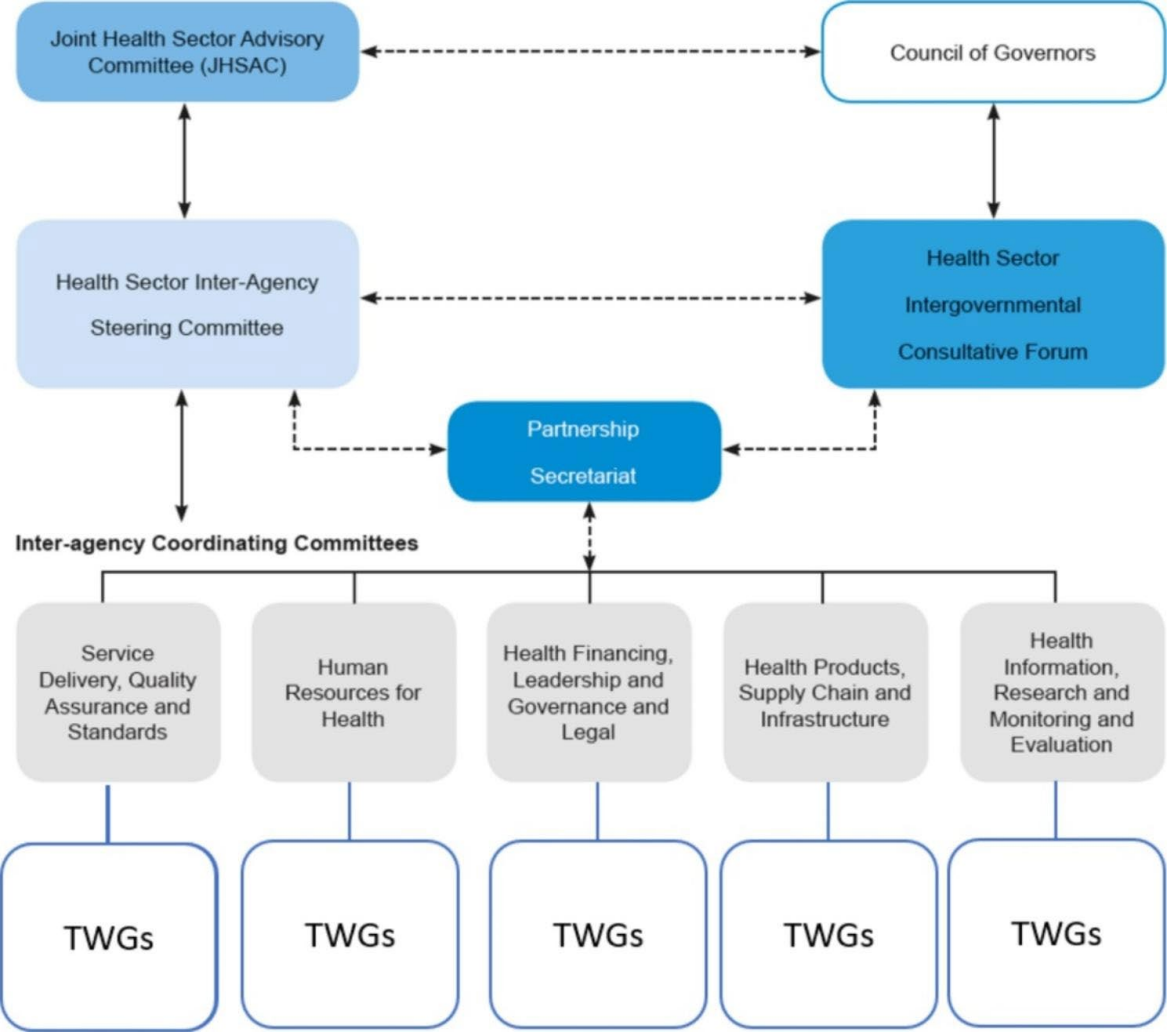
Kenya Health Sector Partnership and Coordination Framework 2018–2030. Source: adopted from the Kenya health sector strategic plan 2018–2023

The framework outlines structures that bring together and coordinate actors at different levels of the health system (Fig. 2). The highest level of the framework is the Joint Health Sector Advisory and Oversight Committee (JHSAC) whose role is to provide overall leadership and governance of the coordination framework. The JHSAC is chaired by the Cabinet Secretary for Health and co-chaired by the Chair, Council of Governors’ Health Committee, and the chair of the donors for health, Kenya (DPHK). The DPHK is the coordinating structure among donors in Health in Kenya and comprises 15 members that are representatives of the major donors in health in Kenya. The membership of the JHSAC includes the Chief Administrative Secretary (deputy to the Cabinet Secretary), the Principal Secretary Health, the MoH Director General, a representative of Principal Secretary of the National Treasury, and representatives from donors, Non-Government Organizations (NGOs), Faith Based Originations (FBOs), and private sector.

The Health Sector Interagency Steering Committee (HSISC), a network-type mechanism, is the second level in the structure and is comprised of representatives of key health sector actors and provides technical level coordination of actors (government, donors, private sector, civil society, national and county governments). The Inter-agency coordinating committees (ICCs), a network-type mechanism, reports to the HSISC and are the technical arm of the framework that should provide a platform for joint planning, coordination and monitoring of sector activities. There are five ICCs aligned to the health systems building blocks that are led by technical heads from the MOH and comprise of representatives of stakeholders of the specific health system function. The ICCs are expected to establish Technical Working Groups (TWGs) on specific sub-themes to undertake specific assignments on an ad hoc basis.

The partnership and coordination framework also provides for the Kenya health sector intergovernmental consultative forum, an annual event for all sector actors to review performance, engage, and identify priorities for the coming year. Finally, the framework provides for the MOH, CDOH, and health sector partners to establish additional coordination structures that are aligned to the official framework based on need.

### Health sector coordination at the national level in practice

**The network-type coordination mechanisms prescribed by the Kenya health sector partnership and coordination framework described above were established and operational at national MOH level.** Specially, the inter-agency coordinating committees (ICCs) and technical working groups (TWGs) were reported to be operational.

“*There is a framework by program area where coordination takes place at the national level through the interagency coordinating committees and the new health partnership coordination framework within the MoH*” (Respondent 7, national level).

The framework structure was reported as a useful mechanism for strengthening coordination among health sector partners.

**There was duplication in the structures set up to improve co-ordination at the national MOH**. Study respondents felt that there was some duplication between the coordinating structures established by the health sector partnership and coordination framework, and intergovernmental coordination mechanisms. Specifically, it was reported that there were instances of duplication of actions by the ICCs and the health sector interagency coordinating committee. This arrangement led to multiple meetings with the same actors which was considered wasteful.

“*You find a structure that is established as a technical thematic committee under the intergovernmental structures is almost like the one established as an interagency coordinating committee under the partnership framework. Sometimes you find that the two deal with similar things, but they have to remain separate because the intergovernmental structures are government structures and are decision-making structures, while the coordination and partnership structures may be more of advisory in nature to inform the decisions that the government structures make. Sometimes the players find it repetitive when they have to hold a different meeting for the partnership structure and the intergovernmental structure and yet they are discussing the same things.”* (Respondent 6, National level).

**Within the MOH, it was reported that the roles and functions of organizational units was inadequately articulated**. This led to a lack of clear job descriptions for staff that resulted in duplication of roles and responsibilities at the MOH. This was exacerbated by a high turnover of staff in positions and inadequate hand-over between staff when role reassignments occur. Inadequate clarity over staff roles was said to result in duplication of activities, contestations among staff, and delays in decision making, negatively impacting staff performance.

“*There are no clear-cut definitions of what you’re supposed to do. You just do the roles as they come. One day you’re a procurement officer, another day you’re an accountant then you are an engaging at an international level. Also, there was no handing over. I came from a health facility to this office and therefore needed some sort of orientation as regards the job description. But when I landed there, I was told, this is your office. And I wondered, so what exactly am I supposed to do? I’m still learning.*” (Respondent 4, national level).

**Functions were duplicated between various MOH units and vertical disease programmes (HIV/AIDS, TB, Malaria, family planning Immunization) and among the vertical disease programmes**. For instance, while there was an MOH unit responsible for the monitoring and evaluation of health programmes, each vertical programme had a monitoring and evaluation unit. Study participants felt that monitoring and evaluation was a cross-cutting function, and that this duplication led to wastage of resources.

*“Within the MoH, for example, there is the M&E unit for the division of M& E. But if you go to the various programs, divisions, etc, each has their own M& E unit hence duplication of this function*.” (Respondent 5, National level).

The duplication of the monitoring and evaluation function was also related to the fragmentation of health information systems among vertical programmes and between these programmes and the non-vertical operations of the MOH. This was reported to duplicate the effort and data collected leading to resource wastage and increase in the reporting burden of health facilities with negative implications for service delivery and quality [37].

**Supply chain for essential commodities was fragmented across vertical programmes, and the non-vertical health operations of the MOH.** This was caused by fragmented funding sources for vertical programs. For example, while the Kenya Medical Supplies Agency (KEMSA) handled the procurement and supply health commodities in the public health sector, procurement for vaccines funded by GAVI was handled by UNICEF, procurement for donor funded family planning commodities was handled by UNFPA and CHAI, and the procurement for some malaria commodities was handled by the United States Government (USG) [37]. Further, while storage and distribution of all health commodities was handled by KEMSA, the immunization programme operated a parallel distribution system with its on central stores, regional and sub-national depots [37].

**Participants also reported fragmented coordination of functions between the MOH state agencies that had health sector regulatory functions**. It was noted that health facilities were subjected to multiple assessments and regulatory requirements from multiple health regulatory agencies, that were sometimes overlapping.

“*Some SAGAs have had conflict within the counties. For example, recently we got a circular that the Kenya medical practitioners and dentist council (KMPDC) is the only organization that should be regulating facilities. Then shortly, we saw another memo saying the Kenya health professionals oversight authority (KHPOA) is the one that should regulate. The National Cancer Institute is also supposed to regulate cancer treatment centers. The National Health Insurance Fund (NHIF) is also regulating. There’s quite a bit of confusion and hence need for harmonization of this area. The facilities have been complaining that it is too much, every other person is coming with a requirement and requiring them to pay*” (Respondent 9, National level).

### Health sector coordination at the county level in practice

**While the CDOH had formal organizational structures in place, these sometimes had inadequate role clarity and overlaps in both counties**. This resulted in duplication of efforts as well as interference of duties which negatively influenced performance. Participants reported that this duplication of roles was worsened by the creation of multiple similar or related roles. For instance, counties would create more than 2 chief officer, or director of health services positions with overlapping roles to accommodate political interests. This was seen to influence health system performance negatively.

“*There is a lack of clear demarcation or definition of who’s supposed to do what, and who should supervise the other because devolution has created a lot of confusion. Positions such as the CEC Member were created to manage the departments and they may not understand how things should work and end up in interfering with how facilities are run, how supplies are made, or how transfers are made. Sometimes they end up doing work that they’re not supposed to do. (County Referral Hospital Manager, County B)*

“*You need to have one structure across the country. Some counties have several chief officers within the health department but here we have one. A unified structure improves efficiency*” (County Manager 1, County B).

**Planning and budgeting processes at the county level were misaligned.** Specifically, priorities that were included in the plans, and the resource requirements did not match those that were included in budgets. This resulted in work plans that had higher budgeted amounts than the available resources.

*“I think one of the challenges of the health sector is that some people develop work plans worth more than the available resources. It’s a historical problem. If we truly change that, then we say that these are the resources that we have for this financial year.”* (Respondent 5, National level).

**CDOHs experienced frequent leadership changes, coupled with inadequate mechanisms to ensure continuity of initiatives over time in both counties.** As a result, newly installed CDOH leaders often had to re-start initiatives, which was thought to be disruptive and impact negatively on the co-ordination of the county department of health activities.

“*There’s a high turnover, particularly at the political leadership level. If you look at the CEC Member and the Chief Officer, they are key people in terms of the running of the departments. From one government to the other, there is an obvious change. That affects the department’s previous vision and the plan and that affects the service delivery*.” (County Manager 2, County B).

“An *example is the reproductive health policy, the county executive of health (CEC) led the development of a draft, but when a new county executive was appointed, that draft thrown away and the development of a new document was started*” (County Manager 1, County B).

**In both counties, study participants also reported that there was fragmented coordination of service delivery across neighboring counties**. It was noted that county residents would often seek care in other counties. Also, events such as disease outbreaks often had cross-border origins and impacts. However, counties often planned their interventions independently with little/no consultation among them. This was corroborated by the health sector medium term review (MTR) which found reported complexities in managing engagements with counties owing to difficulties in getting timely consensus across all 47 counties poses coordination dilemmas. The difficulty in obtaining consensus among 47 independent counties has posed challenges for coordination. This resulted in uncoordinated service delivery that compromised the overall effectiveness of individual county plans.

“*Sometimes you find that there’s an outbreak in one county, and that county depends on the neighboring county for some health services and yet the two counties do not coordinate outbreak response. To address the outbreak properly, you need to coordinate with the other county so as to intervene at the source of the outbreak. However, without coordination, a neighboring county cannot intervene because it is outside their jurisdiction. There needs to be more collaboration at technical level between neighboring counties.*” (County Manager 2, County A).

“*We need to have more collaboration with our neighboring counties. Once we understand what is happening there, we can do the right thing here. If you look at interventions currently, everyone is planning alone. There should be a more organized way of inter-county coordination particularly for epidemics. For example, a cholera transmission source could be within their area of settlement but you’re receiving patients here for treatment. In order to address the outbreak properly, you need to coordinate with the other county because you cannot intervene at the source because it’s a different jurisdiction. There needs to be more collaboration at technical level in a platform.*” (Sub-county Manager 1, County B).

**In county B, the was fragmented coordination between the county health management teams (CHMTs) and the sub-county health management teams (SCHMTs)**. This was attributed to the inadequate role clarity of the sub-county teams, and inadequate role demarcation between the CHMTs and SCHMTs. As a result, sub-county teams were sometimes not included in county level planning for key functions such as procurement and finance and bypassed by county teams in the implementation of health programs. This was thought to limit the effectiveness of the sub-county health management teams in supporting local implementation of activities and hence compromise service delivery.

“*Between the county and the sub counties, there are no clear-cut responsibilities and roles for the staff. Sometimes, the county may assume that the sub-county teams have done the work, but the sub county teams are not aware. This leads to delay of service delivery. And then there is no component of ownership. The sub county will not honor those projects which are thought to be for the county level*.” (Sub-County Manager 2, County B).

“*Sub-county teams are kind of delinked from procurement in our county. We don’t undertake procurement. All we do is to do the request. We are often in darkness. This tends to lower the activities which are supposed to be undertaken at the sub-county level, some of the things are delayed or even not attended to which affects the service delivery to the public.”* (Sub-County Manager 2, County B).

The lack of coordination between CHMTs and SCHMTs was said to reduce the motivation and ownership of health programs by sub-county managers.

*“Before devolution, we used to have authority to incur expenditure expenditure at sub-county level. Since devolution, we at the sub county level, we are totally in darkness. We don’t know what is happening in terms of procurement, or anything concerned with financing. We are really demoralized. Imagine a situation where we are supervising people at the facility level who have authority to incur expenditure, yet we at the sub county level we don’t have at all. What is the logic of having the sub-county? Then we can just have one structure county, county health management team and then forget about sub counties*.” (Sub-County Manager 3, County B).

### Coordination between the National and County level

An MOH medium term review (MTR) of performance found that the coordination between the national and county governments had improved since the inception of devolution, enhanced by the Council of Governors. However, coordination between the two levels of government was constrained by inadequate funding of coordination activities. The MTR also reports inadequate stakeholder mobilization, engagement, and communication by the national government and the counties [32]. For instance, while the health sector strategy targeted to hold 4 intergovernmental consultative forums in a reporting year, it held none in 2017/18, and only 1 in 2019/20.

**Study participants from both counties reported instances of misalignment between national MOH and County government functions and priorities**. This was said to take the form of the usurping of county government roles by the national government and resulted in the misalignment between programmes implemented by the national government and local priorities. For instance, under the medical equipment scheme (MES) the national government leased and installed a standard set of selected medical equipment in public hospitals in each of the 47 counties. This was despite some counties having already purchased and installed some of this equipment and hence leading to wastage. Further, it resulted in the underutilization of the equipment due to lack of complementary inputs at the health facility level such as proper power connection, and skilled staff to operate the equipment.

“*The famous MES project by the national government led to equipment lying in the facilities without being utilized. Such equipment was delivered without formal consultation with the facilities and they ended up as white elephant projects. For example, some equipment required a three-phase power supply and the power supply in those areas was not three-phase*.” (County Manager 1, County B).

**Study participants from both counties reported duplication of functions between the county department of health and vertical programmes.** Specifically, there was duplication of functions and staff roles between county department of health staff, and staff employed by vertical disease programs. This duplication was thought to result in wastage and reduce accountability for performance among staff with overlapping roles.

“*Part of the problem is that many of those vertical programs have their own officers in the counties implementing them. Is that efficient in terms of prudent use of financial resources or would it be cheaper if we had programs of different donors being implemented by officers of the county government and then we provide effective supervision and things like that? Questions of accountability come in when we have so many of these programs standing alone*.” (Respondent 6, National level).

***“****There is usually some overlap, for example, there is a role the county will play, there is a role Malaria Control will also play. Training can be done by either. Sometimes even in terms of supplies, the county may procure sometimes occasionally. It’s not really defined that county can only supply the drugs while the program should do this activity*.” (County Manager 1, County A).

Vertical programs also implemented activities independently with minimal coordination with county government implementation activities. Supportive supervision was one such activity where vertical programmes went directly to health facilities bypassing the counties.

**There was misalignment between the policy formulation role of the national government and county government implementation role.** It was reported that the national government would sometimes formulate policies without proper consultation with the counties. This resulted in poor implementation of policies at the county level. It also resulted in delays in implementation due to multiple iterations of post-hoc consultations between the national government and counties.

“*The other grey area is policy and legislation development which is the mandate of the national government. However, we (Counties) argue that they are autonomous entities that should also be properly consulted and engaged because we will be responsible for their implementation. We need to ask ourselves who is following up on the enforcement of these policies at the county level.”*, (County Manager 3, County A)

“*There are policy aspects where the national government still treats us like a mother and child relationship. They develop policies without engaging us and then call us to rubber-stamp it when we’re launching*.” (County Manager 1, County B).

“*Policies and legislations are originated by MOH and then sent to the COG which brings about inefficiency. It would be better if both levels of government were to brainstorm together and agree at the draft stage. This would cut down on the time of going forth and back to agree on a draft legislation. There is also the tendency for parties to feel this is an imposition by one level of government eliciting a reaction of rejections from the counties*” (Respondent 8, National level).

Additionally, lack of proper policy/legislative coordination mechanisms between the two levels of government negatively impacted on the accountability for implementation. It was not clear how nationally formulated policies would be enforced in autonomous counties.

“*The other grey area is the policy and legislation area which is the mandate of the national government. Co-ordination of technical issues between the two levels has been a challenge. Counties argue that they are individual entities. We need to ask ourselves who is following up on the enforcement of these policies at the county level? At one point, it was proposed that the county directors of health should report directly to the DG at the national level. That was repulsed by the governors as a breach to county autonomy”* (Respondent 7, National level).

**The national government role in in-service training of health workers was misaligned with the county government role in recruiting and managing health workers.** Specifically, while the national government would fund the in-service training of health workers, these staff remained on the payroll of county governments. Both counties found it challenging to recruit additional staff to provide cover for staff on training study leave because of constrained resources and capping of staff payroll expenditures. This compromised the input mix of counties, and compromised service delivery.

*“We have to address the human resource training issue. We need proper coordination between the national and county governments to make sure that when health workers go for training, counties are not left burdened by the financial responsibility of paying their salaries. Since training is a national government responsibility, they should meet the cost of salaries for staff in training. This will free up resources for counties to pay for replacement staff”* (County Manager 2, County A).

“*Why does the national level have 9 billion allocated for two functions; policy and training yet counties have to pay salaries of those released for training and find their replacements? We need a proper coordination with the national government to make sure that salaries of the health workers who have been released for training is picked up by the national government because counties are currently overburdened by the financial constraints*.” (County Manager 1, County B).

### Coordination between the health sector and donors

**While the partnership framework was established at the national level, the MTR reported that it had not been fully adopted at the county level in both counties**. In addition to the structures prescribed by the partnership framework to coordinate partnerships at the national and county level, county governments in addition had established County Health Stakeholders Fora as the coordination mechanisms at county level. Several challenges were however highlighted by study participants. Counties also enhanced coordination by engaging and including donors in the development of the county department of health’s annual work plans. The MOH at the national level and county departments of health also had an office and staff designated to provide liaison and coordination between donors and the government. These were thought to have increased co-ordination of development partner activities and reduced duplication.

“*We have a stakeholders’ forum where we bring on board all partners in order to understand what they’re doing. For example, HIV could be an emerging issue, but the partners are largely skewed towards addressing maternal health. It becomes difficult to for them to support in that area even if it is your need. You may have adequate numbers supporting you in one area but lack partner support in another area. We try to know them and help them understand what we expect from them so that then they contribute towards what we want*” (County Manager 2, County B).

“*We have the office of the liaising officer which coordinates the activities of the partners*” (County referral hospital Manager, County A).

“*What is helping us is collaborative development of our annual work plans. It is clearly spelt out which activities will be supported by the County Government and those that will be supported by specific partners. If you look at our annual work plan, you will see each activity indicated and costed and the entity responsible.”* (County Manager 1, County A).

**However, study from both counties participants noted that there were instances where county priorities were misaligned with donor priorities**. This was because some donors would develop their programs of work in consultation with the national government and with inadequate or no consultation with counties and their health facilities.

“*They (donors) don’t look at the priorities that we have at the sub-county or facility level. They come with their priorities and impose them on you. Before any development partner comes on the ground, they should give us a chance to discuss with them our priority areas so that they fit in our plans and not us in their plans.”* (Level 4 health facility manager, County A).

*“It would be better if we at the facility were involved in forums where we discuss the gaps we have at the ground level. You find that a donor has come to support an area that’s maybe not a place of need. We often feel like we are left out because we are not involved in the decisions regarding which areas need to be supported. You find that a donor has been sent and you’re told that the donor is coming to do something which was pre-determined I don’t know by who. We are not involved.”* (Level 3 Health Facility Manager, County B).

**Some development partner activities were duplicated**. Donors would sometimes support and carry out similar tasks while other priorities remained neglected. This was attributed to the inadequacy of the partners coordination framework and was thought to lead to wastage of health sector resources.

“*The partners are not well coordinated. For example, if you look at about 10 partners here, they are haphazardly doing the same thing. Sometimes even we at the county are also doing the same thing. That is a waste of resources*” (County Manager 1, County B).

### Coordination between the health sector and other sectors

**There were inadequacies in the coordination of programmes and activities between the health sector and other sectors**. This was thought to compromise the delivery of services that were dependent on the actions of other sectors.

“*We need to have proper coordination between health department and other departments such as the water department because a facility cannot run smoothly without water. For example, the last two weeks, we had a lot of problems at one of our rural dispensaries because there was a breakdown of borehole that was serving the area there, and therefore the facility was not able to get water.”* (County Manager 1, County B).

“*For non-communicable disease space where we are affected by sectors beyond health, there is need to look at co-ordination with others beyond the health sector. For example, improved physical activity for the populace would require a good road network with consideration for running and cycling. This is because the risk factors for cancer span beyond health sector and 40% of cancers can be prevented through risk factor reduction.”* (Respondent 9, National Level).

## Discussion

This study examined health sector co-ordination in the Kenyan health sector. The study found that the Kenyan health sector has formal coordination mechanisms that are characterized by both hierarchical and network-type features. We found that these coordination structures are more established at the national level, but less so at the county level. Further, even where the structures formally exist, the study found coordination gaps in the form of duplication, fragmentation, and misalignment of health system functions and actor actions in line with the study’s conceptual framework. Inadequate coordination in the Kenyan health sector appears to be driven by three interrelated factors. First, there is an underlying weakness of governance systems in the health sector. This partly explains the lack of formal organograms at the national MOH, fragmented functions of MOH agencies such as regulatory agencies, and poor role clarity among staff at the MOH and county DOH. Related, and perhaps amplifying the underlying governance challenges, is the Impact of rapid devolution of the Kenyan health system, creating new governance arrangements that have taken time to develop the capacity for effective coordination. Rapid devolution has been cited as the reason for weak coordination between national and county governments [38], and between county health management teams and sub-county health management teams [39]. Third, while, on paper, the health sectors engagement was guided by SWAp principles. In practice these principles were violated. The role of donor funding in fragmenting funding, priorities, and implementation efforts contributes to weakening coordination. Donor funding arrangements are responsible for fragmented supply chains for essential health commodities, and the siloed operations of vertical disease programmes, and duplication in donor implementation. This is consistent with what has been observed in several LMICs where prolonged donor dependence undermines health sector coordination [11].

As anticipated by the study’s conceptual framework, the study found that the coordination gaps identified are likely to compromise the efficiency of the Kenyan health sector in several ways. One potential pathway is by affecting the transaction costs of health system functions. Transactions costs were affected by duplication and/or fragmentation of actions by health system actors at both the national and county level, as well as across levels of government. Within government, duplication was identified in the coordination structures, inadequate role clarity among staff, as well as between the MOH at the national level, or the CDOH at the county level and vertical disease programmes. Duplication was also observed among donors. This led to wastage of health sector resources. The fragmentation of supply chains of essential health commodities across vertical programmes, M&E systems, and the regulatory function of the national MOH were also thought to increase transaction costs. Fragmentation of supply chains for essential commodities and monitoring and evaluation systems has been shown to result in wastage of human and financial resources and contributing to administrative inefficiencies [10, 11, 40, 41]. The duplication of health sector activities has been also been shown to *lead* to the dilution and distortion of limited human and financial resources [11, 12].

A second pathway through which health sector coordination may influence the efficiency of the Kenyan health system, which was also anticipated by the study’s conceptual framework, is by compromising the effectiveness of implementation and hence performance of health systems. One way in which health system performance was likely influenced is through reduced staff motivation occasioned by poor role clarity at both the national MOH and county department of health. The fragmentation of functions such as supply chains, planning and implementation between the county and sub-county level, and between counties was also thought to compromise the effectiveness of implementation of health programmes. Similar findings have been shown elsewhere where fragmented health systems delivery has contributed to difficulties in access to care, poor technical quality, and discontinuity of care [11, 12, 42, 43]. Further, fragmented donor activities have been shown to not only be costly but also challenge the effective implementation of health interventions [12, 37, 44, 45].

Another way that poor coordination affects health system performance is by compromising health sector accountability, also anticipated by the study’s conceptual framework. Accountability was compromised when health system functions were duplicated or fragmented and could lead to poor implementation of health programmes. For instance, fragmented roles of local governing bodies has been shown to impede monitoring and accountability of district level health partnerships and undermine national structures and systems [46]. Further, misalignments of policy formulation and priorities between the national government and county government, between the government and donors, and between the planning and budgeting processes in the health sector was also thought to compromise the implementation of health sector programmes.

We did not find systematic differences in coordination arrangements between the county that was ranked as efficient and the one that was ranked as inefficient by the quantitative efficiency analysis. This could be because we were not able to fully capture the variation in coordination practices between the counties: differences may have been in terms of intensity rather than occurrence and hence are difficult to measure using a qualitative approach. It could also be because the counties that were ranked as efficient by the quantitative analysis by being on the efficiency frontier are inefficient in absolute terms, even though they are relatively more efficient than the counties that are at a distance from the frontier. Further, the vertical coordination mechanisms that are managed centrally (at the national level) are critical, and affect both counties equally, making it difficult to tease out differences in coordination across counties.

This study collected data from two out of 47 counties may limit the generalizability of the study findings to the entire country. However, given that coordination mechanisms do not differ widely among the counties, the results obtained in the study could provide useful insights to other counties. Another limitation of the study is the failure to include sub-county respondents from county A. While this limitation is partially mitigated by the eliciting of perspective about sub-county from the other respondents, who are typically well versed with the operations of all levels, collecting views from this level in county A would have strengthened the study. The study would have further been strengthened by collecting data from actors outside of the health sector including the treasury department and county assemblies.

The study highlights several potential policy levers for improving health sector coordination in the Kenyan health sector that could improve health system efficiency. First, the national and county governments should review and align the Kenya health sector coordination framework with the intergovernmental coordination mechanism to eliminate the duplication identified in these two mechanisms. Second, the national and county governments should strengthen the implementation of the Kenya health sector coordination framework at the county level. This will entail the communication and sensitization of counties on the framework, the establishment of coordination structures at the county level, and supporting its implementation at this level by among others adequate resourcing. Third, the national and county governments should strengthen mechanisms for strengthening donor coordination in the health sector, in line with SWAP principles. Options to explore include the introduction of common funding approaches such as basket funds that have been shown to enhance coordination elsewhere [47, 48]. Fourth, the MOH and county government should develop and implement mechanisms to integrate vertical disease programmes with the rest of the health system. This includes key functions such as procurement and supply chain, financing, monitoring and evaluation, and service delivery. Experience from other countries has shown that such integration improves health systems efficiency [49]. Fifth, the MOH and county governments should review internal organizational structures to enhance role clarity in the administrative units and staff leading these units. This will review the development of clear organograms, and explicit role assignment across the units in the organogram. The organograms and their functional assignments will also need to be effectively communicated and accessible to staff at the national and county level, and partners working with the MOH and CDOH. An effort to harmonize organizational structures of county departments of health across counties should be explored. Sixth, there is a need to review and enhance the clarity of functional assignment between the national and county governments in the health sector. While this clear in the Kenyan constitution, the coherence of its operationalization has not always been clear. Seventh, counties should consider initiating health sector coordination mechanisms between counties to reduce the fragmentation of health system functions across neighboring counties. An example is the inter-county, multi-stakeholder human resources for health coordination mechanism that have been initiated to coordinate human resource for health across neighboring counties in Kenya [50].

## Conclusion

This study examined health sector coordination and its influence on the efficiency of the Kenyan health system. The study found that while formal coordination structures exist, duplication, fragmentation, and misalignment of health system functions and actor actions compromise the coordination of the health sector. These coordination challenges appear to be driven by weak governance, the effect of devolution, and the fragmentation caused by donor funding arrangements. These coordination challenges negatively impact on the efficiency of the Kenyan health system by increasing the transaction costs of health system functions and compromising health system performance. The study identifies several potential strategies for strengthening health sector coordination that are likely to enhance health system efficiency.

## Data Availability

The datasets generated and/or analysed during the current study are not publicly available due to participant confidentiality but are available from the corresponding author [EB] on reasonable request.

## List of abbreviations

AIDS: Acquired Immunodeficiency Syndrome
CAS: Chief Administrative Secretary
CDOH: County Department of Health
CEC: Chief Executive Committee Member for Health
CHAI: Clinton Health Access Initiative
CHMT: County Health Management Team
CIDP: County Integrated Development Plan
COC: Clinical Officers Council
COG: Council of Governors
CS: Cabinet Secretary
DEA: Data Envelopment Analysis
DG: Director General
DPHK: Development Partners for Health, Kenya
FBO: Faith Based Organization
GDP: Gross Domestic Product
HIV: Human Immunosuppression Virus
HSISC: Health Sector Interagency Steering Committee
ICC: Inter-agency coordinating committees
JHSAC: Joint Health Sector Advisory and Oversight Committee
KEMRI: Kenya Medical Research Institution
KEMSA: Kenya Medical Supplies Agency
KES: Kenya Efficiency Study
KII: Key Informant Interview
KMHFL: Kenya Master Health Facility List
KMLTTB: Kenya Medical Laboratory Technicians and Technologists Board
KMPDC: Kenya Medical Practitioners and Dentists Council
KMTC: Kenya Medical Training College
KNDI: Kenya Nutritionist and Dietetics Institute
KNH: Kenyatta National Hospital
LMIC: Low-Middle Income Country
M & E: Monitoring and Evaluation
MES: Medical Equipment Scheme
MOH: Ministry of Health
MTR: Mid-term Review
MTRH: Moi Teaching and Referral Hospital
NACC: National Aids Control Council
NCK: Nursing Council of Kenya
NGO: Non-Governmental Organization
NHIF: National Hospital Insurance Fund
PPB: Pharmacy and Poisons Board
PS: Principle Secretary
SAGA: Semi-autonomous Government Agency
SCHMT: Sub-county Health Management Team
SERU: Scientific Ethics Review Unit
SWAp: Sector-Wide Approach
TWG: Technical Working Group
UHC: Universal Health Coverage
UNICEF: United Nations International Children’s Emergency Fund
UNPF: United Nations Population Fund
USG: United States Government
